# NOD1 promotes leukocyte clearance and limits inflammation in female mice during obesity‐associated acute lung injury

**DOI:** 10.14814/phy2.70446

**Published:** 2025-07-04

**Authors:** Rodrigo Rodrigues e Lacerda, Nicole G. Barra, Han Fang, Gabriel Forato Anhê, Jonathan D. Schertzer

**Affiliations:** ^1^ Department of Biochemistry and Biomedical Sciences, Farncombe Family Digestive Health Research Institute, Centre for Metabolism, Obesity and Diabetes Research McMaster University Hamilton Ontario Canada; ^2^ Department of Translational Medicine University of Campinas Campinas São Paulo Brazil

**Keywords:** acute lung injury, inflammation, NOD1, obesity

## Abstract

Obesity is associated with metabolic inflammation, which includes changes to innate immune responses relevant to acute lung injury. NOD1 is a cytosolic pattern recognition receptor involved in sensing bacterial peptidoglycan and has been linked to metabolic inflammation. However, its role in obesity‐associated acute lung injury, especially in females, remains unclear. Here, we investigated the impact of NOD1 deficiency on pulmonary inflammation in female mice subjected to a high‐fat diet and lipopolysaccharide‐induced acute lung injury. Compared to wild‐type controls, obese Nod1^−/−^ mice showed reduced leukocyte and neutrophil numbers in the bronchoalveolar lavage (BAL), but increased BAL levels of TNF‐α, IL‐1β, IL‐6, IL‐17A, and IL‐22, suggesting impaired neutrophil clearance. In the lung tissue, NOD1 deficiency during obesity led to elevated neutrophil accumulation, increased myeloperoxidase activity, reduced CD163^+^ macrophages, and enhanced β‐galactosidase activity. Gene expression analysis revealed upregulation of chemokines, adhesion molecules, and inflammasome components, alongside downregulation of M2 polarization markers. Additionally, obese Nod1^−/−^ mice showed higher NF‐κB and ERK1/2 activation and lower p38 phosphorylation. These findings indicate that NOD1 regulates leukocyte dynamics, inflammation, and macrophage function in the obese lung. We identify NOD1 as a key protective modulator of pulmonary immune responses during acute lung injury under metabolic stress.

## Graphical abstract

NOD1 shapes lung inflammation during obesity‐associated acute lung injury in female mice.

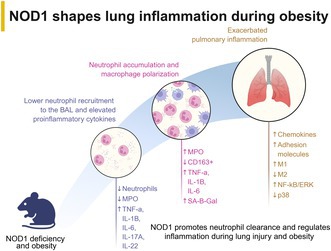

## 1 INTRODUCTION

While obesity is a well‐established contributor to disease severity throughout various organ systems, accumulating evidence suggests that obesity may be protective under specific contexts (Dixon & Peters, 2018; E‐Lacerda et al., 2020; Rodrigues E‐Lacerda et al., 2024). The “obesity paradox” refers to the observation that, in certain lung diseases, individuals with obesity may have a better prognosis than those with normal body weight. These findings underscore the complex and context‐dependent relationship between pulmonary immune responses and metabolic status (Cho et al., 2018; Yao et al., 2023).

Acute lung injury is an inflammatory syndrome that develops in response to both local and systemic challenges. It is marked by the disruption of the lung's endothelial and epithelial barriers, which leads to excessive neutrophil migration and the subsequent release of pro‐inflammatory factors (Matthay & Zimmerman, 2005). The progression of local inflammation, often triggered by infections or trauma, results in increased vascular permeability, edema, alveolar damage, and fibrosis, ultimately leading to loss of lung function. Extrapulmonary causes of acute lung injury include sepsis, pancreatitis, multiple blood transfusions, smoke inhalation, and alcohol and drug abuse (Johnson & Matthay, 2010).

The innate immune system is one of the first lines of defense, which includes the recognition of microbial components through pattern recognition receptors (PRRs). These PRRs can detect pathogen‐associated molecular patterns (PAMPs) and damage‐associated molecular patterns (DAMPs) (Turvey & Broide, 2010). Among these PRRs, the NOD‐like receptors (NLRs) play a central role and can cooperate with Toll‐like receptors (TLRs) to detect different PAMPs and DAMPs to mount immune responses. NOD1, a cytosolic NLR, recognizes γ‐D‐glutamyl‐meso‐diaminopimelic acid (iE‐DAP), a component of the peptidoglycan found predominantly in Gram‐negative and certain Gram‐positive bacteria. Upon ligand recognition, NOD1 recruits the adapter RIPK2 and activates NF‐κB and MAPK signaling pathways, leading to the production of pro‐inflammatory cytokines and antimicrobial peptides (Rodrigues E‐Lacerda et al., 2023). Notably, alterations in gut microbiota composition observed in obesity may influence innate immune signaling by increasing the systemic availability of microbial‐derived ligands, such as peptidoglycan fragments, which can engage receptors like NOD1 (Cuevas‐Sierra et al., 2019).

While LPS is a canonical ligand for TLR4 and does not directly activate NOD1, tissue damage and cellular stress induced by LPS can trigger the release of DAMPs, which are known to prime and modulate NOD1 responses (Chen & Nuñez, 2010; Keestra‐Gounder & Tsolis, 2017). Moreover, LPS exposure compromises mucosal barriers, particularly in obese animals, facilitating the translocation of microbiota‐derived NOD1 ligands into circulation (Clarke et al., 2010). Obesity itself alters gut microbiota composition and increases systemic levels of NOD1‐activating muropeptides (Schertzer et al., 2011), which may enhance pulmonary NOD1 signaling during acute lung injury.

NOD1 mRNA expression is elevated in the subcutaneous adipose tissue of obese mice and humans with metabolic syndrome (Lappas, 2014; Zhao et al., 2011), and NOD1 is elevated in immune cells from individuals with type 2 diabetes (Shiny et al., 2013). NOD1 has been implicated in chronic inflammatory conditions and metabolic disorders, including insulin resistance and obesity‐related inflammation (Ait Yahia et al., 2021; Rodrigues E‐Lacerda et al., 2023; Schertzer et al., 2011). However, despite its established role in metabolic and inflammatory pathways, the contribution of NOD1 in acute lung injury during obesity remains insufficiently investigated.

We have previously shown that NOD2, another cytosolic NLR that recognizes the bacterial component muramyl dipeptide (MDP), protected against lung inflammation in an experimental model of allergic lung inflammation in obese female mice (Rodrigues E‐Lacerda et al., 2024). We were surprised by our previous findings showing that NOD1 deletion during obesity did not significantly affect allergic pulmonary inflammation in a model of OVA‐induced asthma, which primarily involves type 2 immunity and eosinophilic infiltration. However, the current study addresses a distinct type of lung injury: endotoxin‐induced, which is driven predominantly by innate immune responses, characterized by neutrophilic inflammation and type 1/17 cytokine production. These two models differ markedly in their etiology, cellular mediators, and immune pathways (Ci et al., 2012; Domscheit et al., 2020). While LPS and NOD1 are triggered by distinct bacterial components, their signaling pathways converge on shared inflammatory mechanisms (Rodrigues E‐Lacerda et al., 2023). Thus, we sought to determine whether NOD1 plays a more prominent role in this context. Testing NOD1 in this neutrophil‐driven model of acute lung injury allowed us to investigate its function in a mechanistically distinct setting from our previous work and to understand its potential as a context‐specific modulator of lung inflammation during obesity.

Hence, we investigated the impact of NOD1 deficiency on lung inflammatory responses in obese mice subjected to endotoxin‐induced acute lung injury. We found that deletion of NOD1 exacerbates inflammation during acute lung injury in obese mice. This finding may provide new insight into how metabolic dysfunction modulates pulmonary immune responses during acute lung injury.

## 2 MATERIALS AND METHODS

### 2.1 Animals

The McMaster University Animal Research Ethics Board (AREB) approved the procedures of this study in compliance with the Canadian Council of Animal Care's (CCAC) recommendations. We used female Nod1^−/−^ whole body knockout mice from McMaster University's Central Animal Facility (CAF). Knockout mice have been backcrossed more than 12 generations to C57Bl/6N mice; therefore, C57Bl/6N mice (Charles River, cat# 027, RRID:IMSR_CRL:027) were used as wild type controls (WT). Mice were housed in groups of four to five per cage and kept under a 12‐h light/dark cycle. Prior research has used these knockout mice and WT controls (Duggan et al., 2022; Rodrigues E‐Lacerda et al., 2024).

### 2.2 Experimental design

Eight‐week‐old female Nod1^−/−^ mice and C57Bl/6N mice were fed for 10 consecutive weeks with either a control diet (control group) (Envigo, cat# 8640, 17% Kcal of fat) or a high‐fat diet (HFD group) (Research Diets Inc., cat# D12492, 60% Kcal of fat) supplemented with commercially available whole roasted peanut butter without the addition of colorings, sweeteners, and flavorings (Nut, 68.8% Kcal of fat). Acute lung injury was induced by a single intranasal instillation containing 30 μg of lipopolysaccharide (LPS) from *E. coli* O111:B4 (diluted in 40 μL of sterile saline, 20 μL per nostril) (Sigma, cat# L2630), administered 24 h before sacrifice (Man et al., 2021). Before LPS administration, the mice were lightly anesthetized with ketamine/xylazine (50/5 mg/Kg) to avoid LPS being swallowed or expelled. Immediately after administration, the mice were kept with their heads lifted for 1 min to ensure that the entire volume administered entered the lungs. Mice were deeply anesthetized and sacrificed by cervical dislocation, with an intraperitoneal dose of ketamine/xylazine (100/10 mg/Kg).

### 2.3 Metabolic endpoints and bronchoalveolar lavage (BAL) collection

Body mass was assessed weekly to calculate body mass gain. Relative weight of the perigonadal fat depots, serum levels of glucose, total cholesterol (LaborLab cat# 1770080), and triglycerides (Stanbio cat# 2200‐430), and hepatic levels of triglycerides were assessed at the end of the experimental procedure as readouts for obesity. For BAL collection, the trachea was exposed using surgical instruments and cannulated with a 24G catheter. Three sequential flushes of 500 μL of ice‐cold sterile saline were administered into the lungs, and the resultant volume was centrifuged (1000 × *g*, 10 min, 4°C) to separate the pellet cell fraction from the protein supernatant. Supernatants were stored at −80°C for subsequent experiments. Cell pellets were resuspended in 1 mL of sterile PBS and utilized for total cell count in a Neubauer chamber (in Turk's solution) and differential cell count on slides prepared using a cytocentrifuge and stained with Wright‐Giemsa.

### 2.4 Neutrophil myeloperoxidase (MPO) activity

Neutrophil myeloperoxidase activity was measured in leukocytes recovered from BAL fluid (100 μL after resuspension) and in lung tissue homogenates (50 mg/mL). Cells and tissue samples were homogenized in extraction buffer (5 mM K^2^PO^4^, pH 6.0, containing 0.5% HTBA), incubated at 60°C for 2 h to inactivate the endogenous catalase, and centrifuged (10,000 × g, 5 min, 4°C). Next, 10 μL of the resulting supernatant was transferred to a 96‐well plate, followed by the addition of 200 μL of substrate solution (5 mM K^2^PO^4^, pH 6.0, containing o‐dianisidine and 0.0005% H^2^O^2^). The resulting absorbance was measured kinetically at 460 nm (Yu et al., 2006).

### 2.5 Nitrite levels and arginase activity

The concentrations of nitrite and the arginase activity in BAL and lung tissue homogenates were quantified using the Griess Reagent Kit for Nitrite Determination (Invitrogen, cat# G‐7921) and the Arginase Activity Assay Kit (Sigma, cat# MAK112), respectively, following the manufacturers' instructions.

### 2.6 Senescence determination

Senescence‐associated β‐galactosidase activity was measured in lung tissue homogenates using the Senescence β‐Galactosidase Activity Assay Kit (Cell Signaling, cat# 23833), following the manufacturer's instructions.

### 2.7 Cytokine levels

The levels of TNF‐α (BD, cat# 555268), IL‐1β (R&D, cat# DY401), IL‐6 (R&D, cat# DY406), IL‐17A (R&D, cat# DY5390), and IL‐22 (R&D, cat# DY582) were measured in BAL and lung tissue homogenates using commercially available ELISA kits, following the manufacturer's instructions.

### 2.8 Histology and immunohistochemistry (IHC)

The left lung lobe and the left liver lobe were collected and fixed in PBS containing 4% paraformaldehyde for 24 h. Paraffin embedding was performed using an automated tissue processor (EG1160, Leica, Nussloch, Germany). Tissue sections of 5 μm were obtained using an HM325 microtome (Thermo Scientific, Waltham, USA) and dried in an incubator at 37°C for 24 h prior to staining. Hematoxylin and eosin (H&E) staining was performed according to the Manual of Histologic Staining Methods of the Armed Forces Institute of Pathology (Third Edition) (Luna, 1968).

For immunohistochemistry, after rehydrating the tissue sections in a graded ethanol series to water, antigen retrieval was performed in 10 mM sodium citrate buffer (pH 6.0) at 203°F under pressure for 30 min. Sections were permeabilized, blocked with 2.5% horse serum for 30 min, and incubated overnight at 4°C with primary antibodies against MPO (1:1000, Abcam, cat# ab208670, RRID:AB_2864724) and CD163 (1:400, Abcam, cat# ab182422, RRID:AB_2753196). After washing, the sections were incubated with ImmPRESS HRP Horse Anti‐Rabbit IgG Polymer Reagent (Vector Laboratories, cat# MP‐7801) for 30 min. Signal detection was carried out using ImmPACT DAB EqV Reagent (Vector Laboratories, cat# MP‐7801) for 10 min. Slides were counterstained with either Gill's hematoxylin No. 2 or methyl green, dehydrated through ascending ethanol concentrations, cleared in xylene solution, and mounted with Permount. Images were acquired under bright‐field microscopy at 200× magnification using an H600L optical microscope (Nikon, Tokyo, Japan) coupled with a DS‐Qi2 camera (Nikon, Tokyo, Japan). The stained area (%) was quantified as the DAB‐positive area relative to the total tissue area using ImageJ software (NIH, RRID:SCR_003070).

### 2.9 Western blot analysis

Total protein was extracted from lung tissue (100 mg/mL) using lysis buffer (1 M Trizma pH 7.5, 200 mM EDTA pH 7.0, 10% SDS) supplemented with protease and phosphatase inhibitor cocktails. Protein concentration was determined using the Pierce BCA Protein Assay Kit (Thermo Scientific, cat# 23225). Equal amounts of protein were denatured in Laemmli sample buffer containing 5% β‐mercaptoethanol and heated at 95°C for 5 min. Samples were separated on 10% SDS‐PAGE gels (20 μg per lane) and transferred onto PVDF membranes (Millipore, cat# IPVH00010) using the semi‐dry system Trans‐Blot Turbo Transfer (Bio‐Rad) at 25 V for 17 min. Membranes were blocked for 1 h at room temperature in 5% BSA in TBS‐T buffer (20 mM Tris–HCl pH 7.6, 137 mM NaCl, 0.1% Tween‐20), followed by overnight incubation at 4°C with primary antibodies (1:1000) diluted in 5% BSA/TBS‐T. After washing, membranes were incubated for 1 h at room temperature with HRP‐conjugated secondary antibody (1:7000) diluted in 5% BSA/TBS‐T. Protein bands were visualized using Clarity Max Western ECL Substrate (Bio‐Rad, cat# 1705062) and detected with the ChemiDoc Imaging System (Bio‐Rad). Densitometric analysis was performed using Image Lab software version 6.1 (Bio‐Rad, RRID:SCR_014210), and target protein level was normalized to housekeeping protein β‐actin. All antibodies used are as follows: β‐Actin (Cell Signaling, cat# 4970, RRID:AB_2223172), myeloperoxidase (Abcam, cat# ab208670, RRID:AB_2864724), phospho‐NF‐κB p65 (Cell Signaling, cat# 3033, RRID:AB_331284), NF‐κB p65 (Cell Signaling, cat# 8242, RRID:AB_10859369), phospho‐p38 MAPK (Thr180/Tyr182) (Cell Signaling, cat# 4511, RRID:AB_2139682), p38 MAPK (Cell Signaling, cat# 8690, RRID:AB_10999090), phospho‐p44/42 MAPK (Erk1/2) (Thr202/Tyr204) (Cell Signaling, cat# 4370, RRID:AB_2315112), p44/42 MAPK (Erk1/2) (Cell Signaling, cat# 4695, RRID:AB_390779), phospho‐SAPK/JNK (Thr183/Tyr185) (Cell Signaling, cat# 9251, RRID:AB_331659), SAPK/JNK (Cell Signaling, cat# 9252, RRID:AB_2250373), and anti‐rabbit IgG, HRP‐linked antibody (Cell Signaling, cat# 7074, RRID:AB_2099233).

### 2.10 Quantitative PCR

Total RNA was extracted from lung tissue using QIAzol Lysis Reagent (Qiagen, cat# 79306) and homogenized with an automated tissue disruptor (MP FastPrep‐24). RNA purity was assessed by the absorbance ratio at 260/280 nm, with values between 1.8 and 2.1 considered acceptable. One microgram of total RNA, treated with DNase I (Invitrogen, cat# 18068‐015), was reverse transcribed into cDNA using the SuperScript IV Reverse Transcriptase kit (Invitrogen, cat# 18090200). Quantitative PCR was performed on a Rotor‐Gene Q system (Qiagen, Ohio, USA) using the TaqMan Fast Advanced Master Mix (Thermo, cat# 4311806), with 40 ng of cDNA per reaction. The cycling protocol consisted of 50 cycles at 95 °C for 10 s and 58°C for 45 s. Gene expression levels were calculated using the 2^ΔΔCt^ method, with normalization to the geometric mean of the endogenous control β‐actin. The TaqMan Assay IDs (Thermo Fisher, Fremont, USA) for the primers used are as follows: *β‐actin* (mm02619580), *Cxcl1* (mm04207460), *Cxcl9* (mm00434946), *Cxcl10* (mm00445235), *Itgam* (mm00434455), *Itgax* (mm00498698), *Cd40* (mm00441891), *Nos2* (mm00440502), *Ifng* (mm01168134), *Arg1* (mm01190441), *Mrc1* (mm00485148), *Nlrp3* (mm00840904), *Casp1* (mm00438023), *Anxa1* (mm00440225), *Fpr2* (mm00484464), *Casp3* (mm01195085), *Tgfb1* (mm01178820), *Muc1* (mm00449604), *Mmp9* (mm00442991), *Nod2* (mm00467543), and *Tlr4* (mm00445273).

### 2.11 Statistical analysis

Statistical analyses were performed using GraphPad Prism version 9 (GraphPad Software, RRID:SCR_002798). Comparisons between groups (WT control vs. Nod1^−/−^ control, and WT HFD vs. Nod1^−/−^ HFD) were made using the unpaired Student's *t*‐test. All datasets were tested for normality using the Shapiro–Wilk test prior to applying unpaired Student's *t*‐tests. Outliers were identified using the ROUT method with a Q value of 1%. Results were considered statistically significant when *p* < 0.05.

## 3 RESULTS

### 3.1 Impact of NOD1 deficiency on metabolic changes induced by high‐fat diet

**FIGURE 1:**
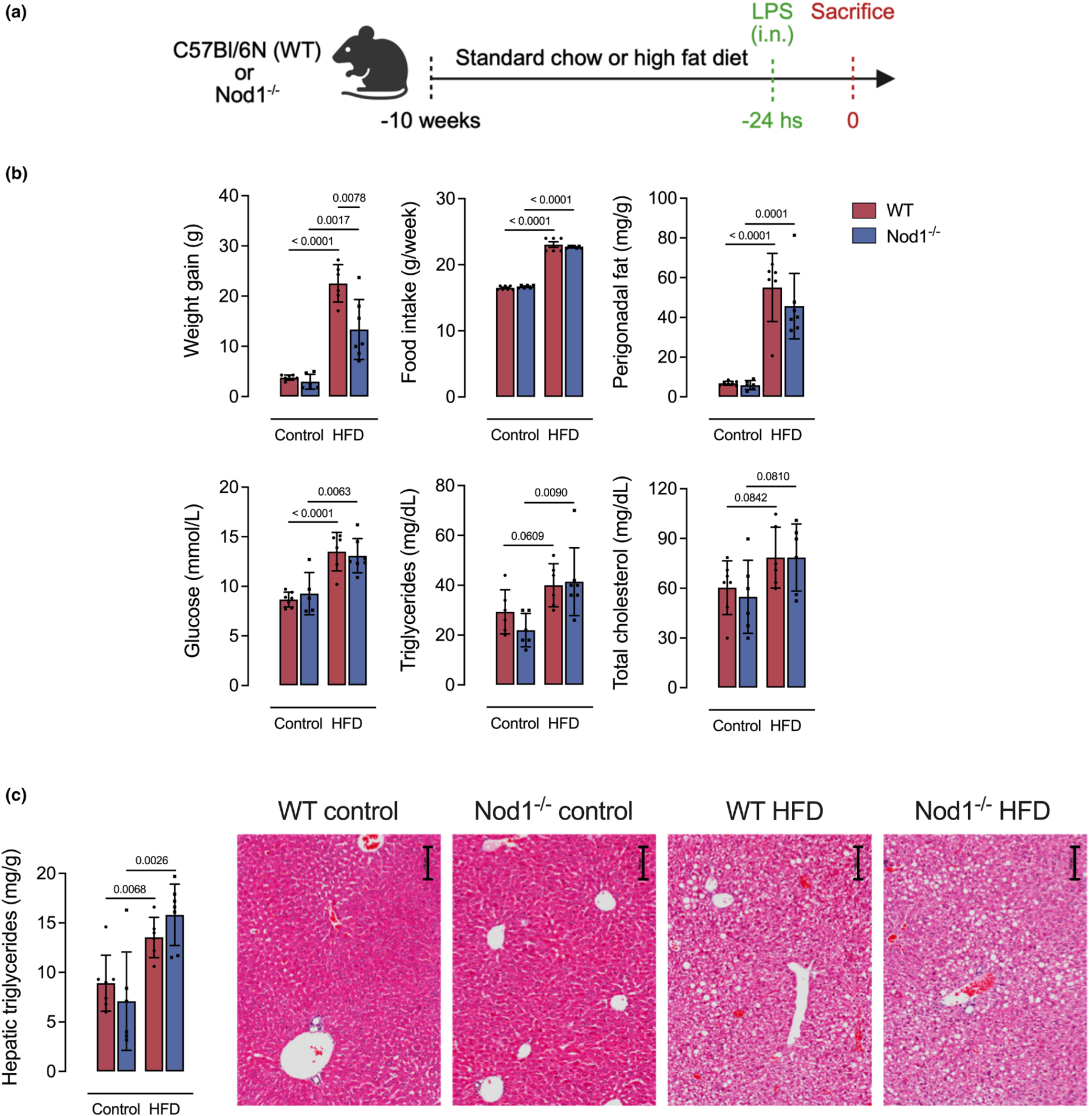
Experimental design and metabolic characterization of WT (C57Bl/6N) and Nod1^−/−^ mice fed either a control or high‐fat diet. (a) Experimental design. (b) Body weight gain, food intake, relative perigonadal fat mass, serum levels of glucose, triglycerides, and total cholesterol. (c) Hepatic triglyceride content and representative images of liver stained with H&E. Data are presented as mean ± SD. Each dot represents one mouse (*n* = 5–8/group). Comparisons were made between WT and Nod1^−/−^ mice within each group (control or HFD) and between WT and WT or Nod1^−/−^ and Nod1^−/−^ using unpaired Student's *t*‐test. Numerical *p* values are shown in the figures for comparisons where *p* < 0.05. WT, wild type. Scale bar, 100 μM.

In response to HFD during the LPS‐induced acute lung injury model (Figure 1a), both WT and Nod1^−/−^ mice exhibited increased weight gain and perigonadal fat pad weight, higher levels of blood glucose and serum triglycerides, as well as higher hepatic triglyceride content, compared to their control diet‐fed counterparts. HFD‐fed Nod1^−/−^ mice had lower body mass gain compared to HFD‐fed WT mice. No significant differences were observed between control diet‐fed Nod1^−/−^ and WT mice for blood glucose and serum triglycerides or hepatic triglyceride content (Figure 1b,c).

### 3.2 NOD1 deficiency exacerbates BAL inflammation and impairs neutrophil recruitment into the BAL during obesity and acute lung injury

**FIGURE 2:**
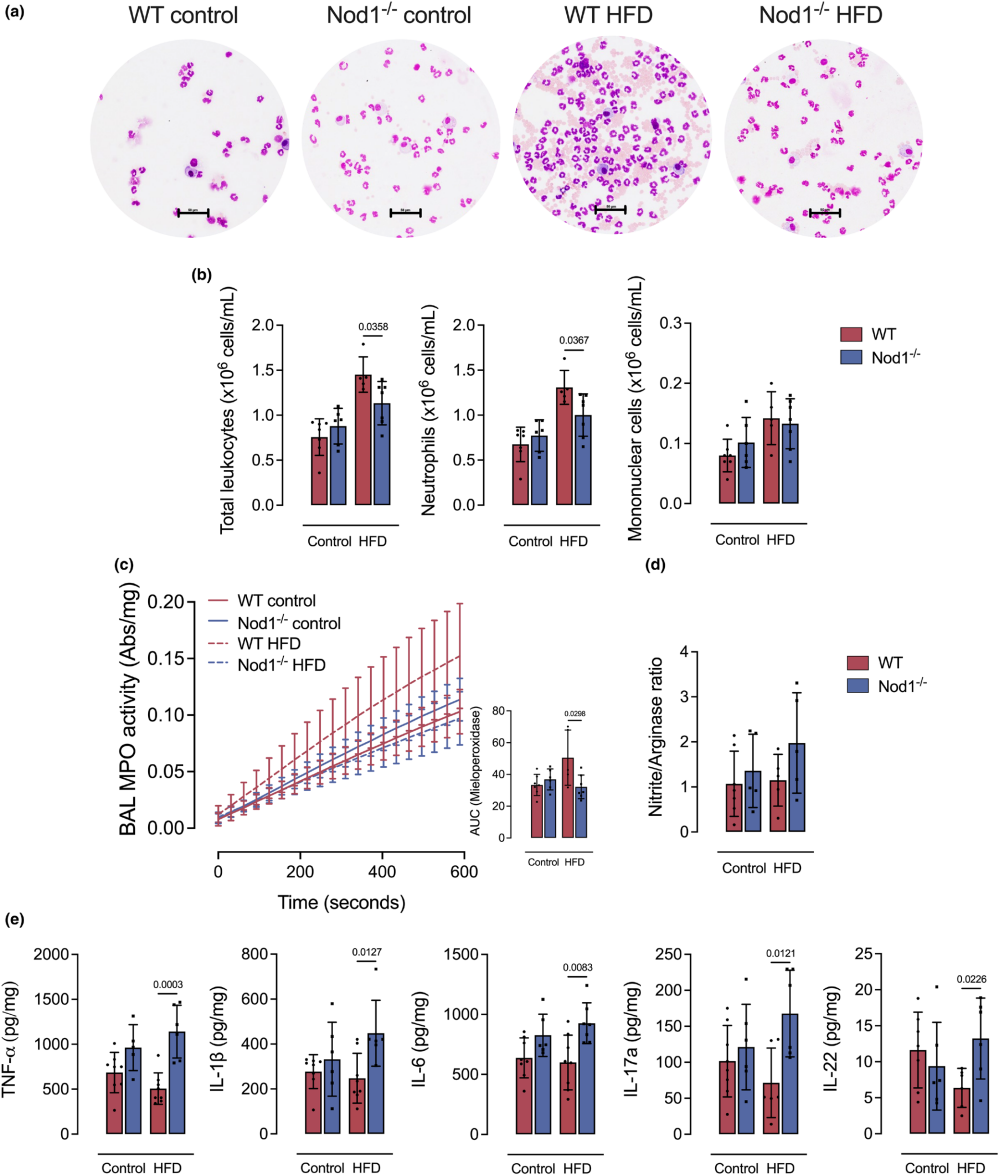
NOD1 deletion impairs neutrophil recruitment and enhances cytokine production in the bronchoalveolar lavage (BAL) during LPS‐induced acute lung injury and obesity. (a) Representative image of BAL fluid collected from LPS‐challenged mice stained with Wright‐Giemsa. (b) Total leukocyte, neutrophil, and mononuclear cell counts in the BAL. (c) Neutrophil myeloperoxidase (MPO) activity as a marker of activation. (d) Nitrite/arginase ratio in the BAL as an index of the inflammatory environment. (e) Levels of TNF‐α, IL‐1β, IL‐6, IL‐17A, and IL‐22 in the BAL fluid. Data are presented as mean ± SD. Each dot represents one mouse (*n* = 5–8/group). Comparisons were made between WT and Nod1^−/−^ mice within each group (control or HFD) using unpaired Student's *t*‐test. Numerical *p* values are shown in the figures for comparisons where *p* < 0.05. WT, wild type. Scale bar, 50 μM.

In response to the LPS‐induced acute lung injury model (Figure 1a), HFD‐fed Nod1^−/−^ mice exhibited a lower number of leukocytes, primarily neutrophils, migrating into the BAL, compared to HFD‐fed WT mice (Figure 2a,b). Neutrophil MPO activity was also reduced in HFD‐fed Nod1^−/−^ mice (Figure 2c). No differences were detected in the nitrite/arginase ratio, regardless of the diet (Figure 2d). HFD‐fed Nod1^−/−^ mice had higher levels of TNF‐α, IL‐1β, IL‐6, IL‐17A, and IL‐22 in the BAL, compared to HFD‐fed WT mice (Figure 2e). No significant differences were observed in leukocyte counts, MPO activity, or cytokine levels in the BAL between Nod1^−/−^ and WT mice under control diet conditions (Figure 2a–e). Thus, NOD1 appears essential for effective neutrophil trafficking into the alveolar space and for limiting excessive cytokine production in the obese lung during acute inflammatory injury.

### 3.3 NOD1 deficiency exacerbates lung inflammation during obesity and acute lung injury

**FIGURE 3:**
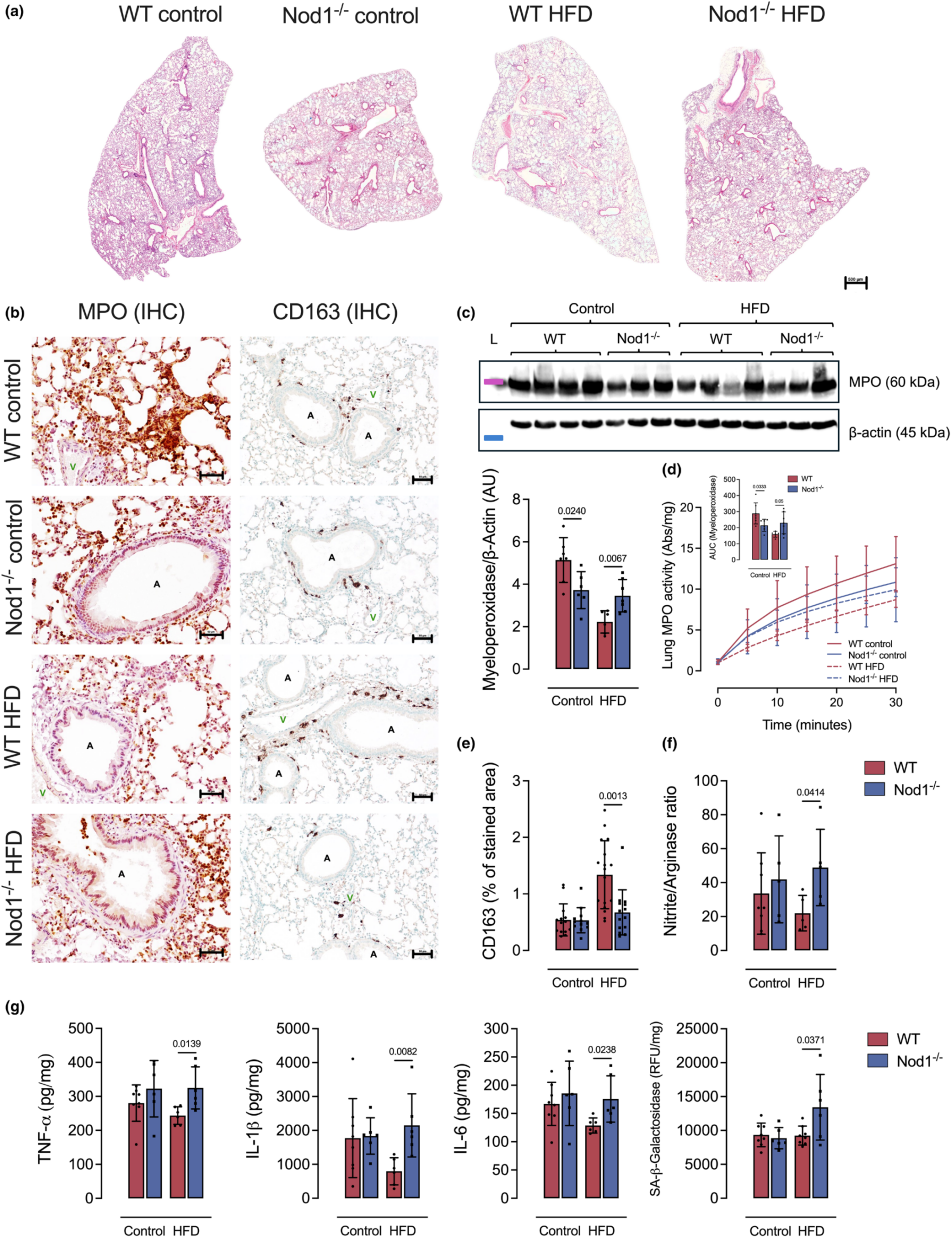
NOD1 deletion exacerbates lung inflammation during LPS‐induced acute lung injury and obesity. (a) Panoramic scan of entire lung sections at low magnification. This overview allows visualization of the overall distribution and extent of inflammatory changes across the lung. (b) Representative immunohistochemistry images showing neutrophil myeloperoxidase (MPO) and the M2 macrophage marker CD163 in lung sections. (c) Quantification of MPO protein expression in lung tissue by Western blot. (d) Neutrophil MPO enzymatic activity. (e) CD163‐positive area in lung parenchyma as assessed by immunohistochemistry. (f) Nitrite/arginase ratio in lung tissue. (g) Levels of TNF‐α, IL‐1β, IL‐6, and SA‐β‐galactosidase activity in lung homogenates. Data are presented as mean ± SD. Each dot represents one mouse (*n* = 5–8/group). Comparisons were made between WT and Nod1^−/−^ mice within each group (control or HFD) using unpaired Student's t‐test. Numerical *p* values are shown in the figures for comparisons where *p* < 0.05. WT, wild type. A = airway. V = vessel. Scale bar, 500 μM (a) and 50 μM (b). Protein ladder (L): 63 kDa (magenta) and 35 kDa (blue).

Compared to HFD‐fed WT mice, HFD‐fed Nod1^−/−^ mice exhibited increased lung inflammation, as evidenced by higher neutrophil MPO expression and activity (Figure 3a–d) and elevated secretion of the cytokines TNF‐α, IL‐1β, and IL‐6 (Figure 3g). This elevated inflammatory profile in HFD‐fed Nod1^−/−^ mice was accompanied by a reduced number of CD163^+^ cells in the lung parenchyma and an increased nitrite/arginase ratio (Figure 3e,f), as well as enhanced SA‐β‐galactosidase activity (Figure 3g). Compared to control‐diet‐fed WT mice, control‐diet‐fed Nod1^−/−^ mice showed reduced neutrophil MPO expression and activity (Figure 3a–d), with no significant differences in the number of CD163^+^ cells, nitrite/arginase ratio, cytokine levels, or SA‐β‐galactosidase activity (Figure 3e–g). These findings suggest that NOD1 is required to limit lung inflammation and prevent excessive cellular senescence during obesity, but not in lean mice.

### 3.4 NOD1 deficiency alters pulmonary inflammatory mediators during obesity and acute lung injury

**FIGURE 4:**
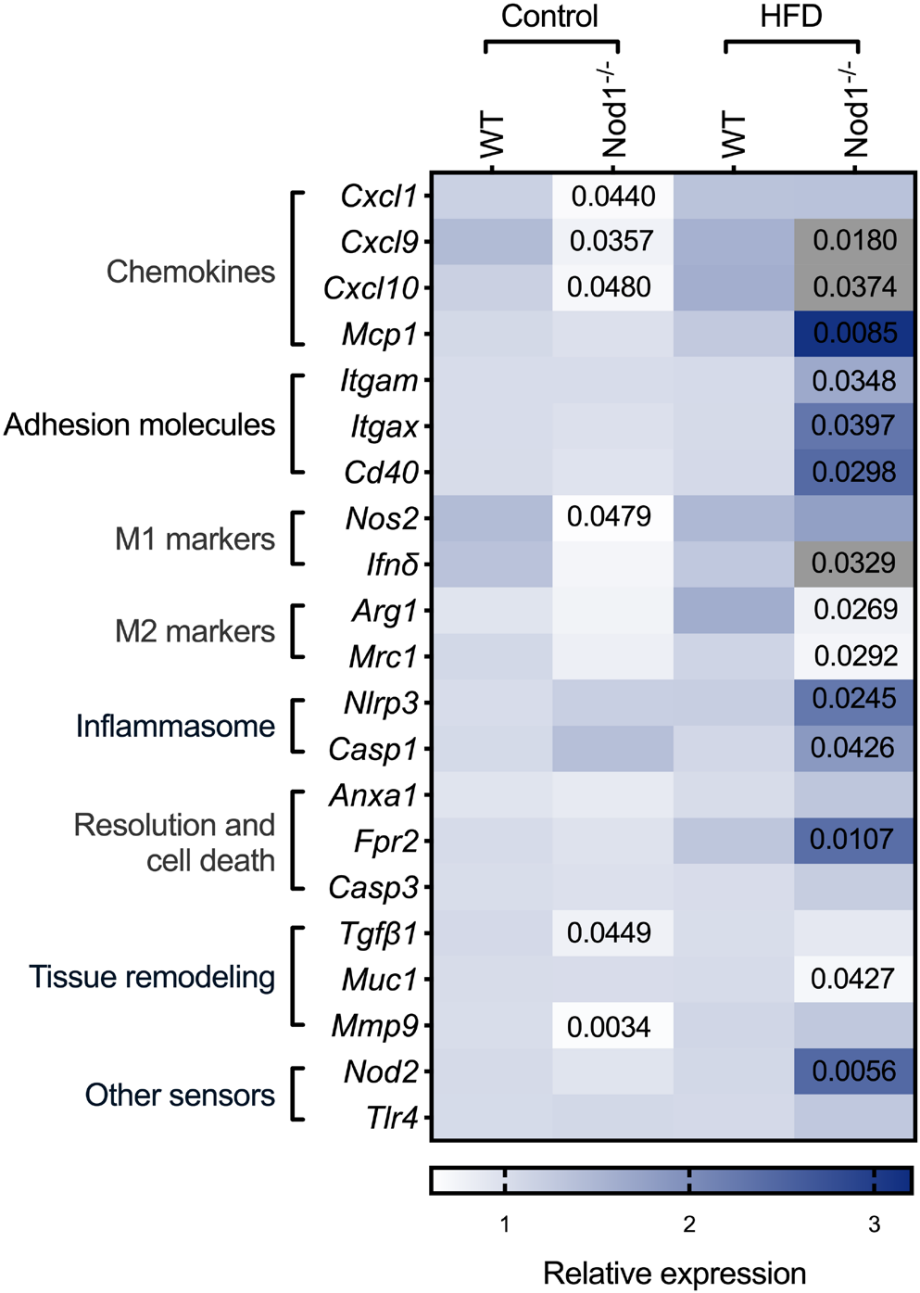
NOD1 deletion alters the pulmonary gene expression profile during LPS‐induced acute lung injury and obesity. Gene expression heatmap of genes related to chemokines, adhesion molecules, M1 and M2 macrophage polarization, inflammasome components, resolution and cell death pathways, tissue remodeling, and other pattern recognition receptors. Data are represented as a heat map showing group averages (*n* = 5–8 mice/group). Comparisons were made between WT and Nod1^−/−^ mice within each group (control or HFD) using unpaired Student's *t*‐test. Numerical *p* values are shown in the figures for comparisons where *p* < 0.05. WT, wild type. Genes shaded in gray represent >3‐fold expression change relative to controls. Gray was used intentionally to avoid distortion of the color scale for the remaining genes, which would have occurred due to extreme expression values.

Compared to HFD‐fed WT mice, HFD‐fed Nod1^−/−^ mice exhibited higher pulmonary mRNA levels of *Cxcl9*, *Cxcl10*, *Mcp1*, *Itgam*, *Itgax*, *Cd40*, *Ifn*δ, *Nlrp3*, *Casp1*, *Fpr2*, and *Nod2*, along with lower mRNA levels of *Arg1*, *Mrc1*, and *Muc1* (Figure 4). In contrast, compared to control‐diet‐fed WT mice, control‐diet‐fed Nod1^−/−^ mice had lower mRNA levels of *Cxcl1*, *Cxcl9*, *Cxcl10*, *Nos2*, *Tgfβ1*, and *Mmp9* (Figure 4). These results highlight that NOD1 protecting against excessive of expression many inflammatory mediators in the lungs during endotoxin‐induced lung injury in obesity.

### 3.5 NOD1 deficiency modulates NF‐κB and MAPK pathways during obesity and acute lung injury

**FIGURE 5:**
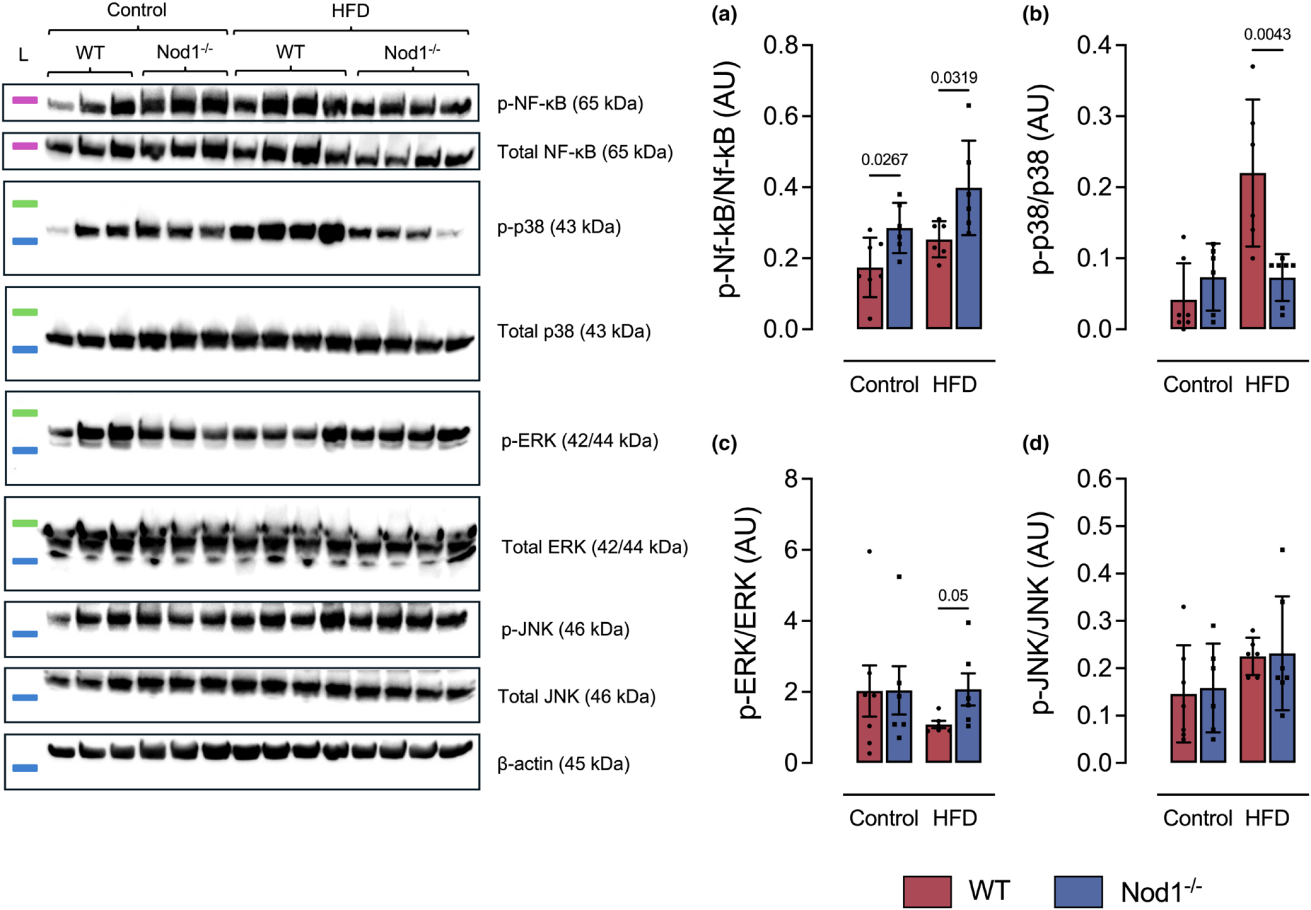
NOD1 deletion enhances NF‐κB and ERK activation while suppressing p38 MAPK signaling in the lungs during LPS‐induced acute lung injury and obesity. Shown are the protein expression levels of phosphorylated and total forms of (a) NF‐κB p65, (b) p38 MAPK, (c) ERK1/2, and (d) JNK in lung tissue. Data represent the ratio of phosphorylated to total protein, as determined by Western blot. Data are presented as mean ± SD. Each dot represents one mouse (*n* = 5–8/group). Comparisons were made between WT and Nod1^−/−^ mice within each group (control or HFD) using unpaired Student's *t*‐test. Numerical *p* values are shown in the figures for comparisons where *p* < 0.05. WT, wild type. Protein ladder (L): 63 kDa (magenta), 48 kDa (green) and 35 kDa (blue).

Compared to HFD‐fed WT mice, HFD‐fed Nod1^−/−^ mice showed higher phosphorylation of NF‐κB (p65) relative to total NF‐κB (p65) and higher phosphorylation of ERK relative to total ERK, but lower phosphorylation of p38 relative to total p38 in lung tissue. No differences were observed in the protein levels of phosphorylated JNK or total JNK (Figure 5a–d). Compared to control‐diet‐fed WT mice, control‐diet‐fed Nod1^−/−^ mice exhibited higher protein levels of phosphorylation of NF‐κB relative to total NF‐κB, but no changes in phosphorylation of ERK relative to total ERK or phosphorylation of p38 relative to total p38 in lung tissue (Figure 5a–d). Full‐length Western blot membranes are shown in Figure S1. These results indicate that NOD1 signaling selectively modulates specific stress kinases and protects against excessive NF‐κB phosphorylation in the lung tissue during obesity.

## 4 DISCUSSION

It was known that obesity can lower inflammation during lung injury. Using both HFD‐fed and genetically obese mice (*db/db*), Kordonowy et al. (2012) showed that after a single dose of LPS aerosolized, these animals exhibited reduced neutrophil chemotaxis into the BAL and lower serum IL‐6 levels (Kordonowy et al., 2012). Similarly, Kuwabara et al., using Wistar rats, demonstrated that an intratracheal LPS challenge led to decreased neutrophil recruitment and cytokine production in the BAL (Kuwabara et al., 2018). Here, we demonstrate that NOD1 is required for lower lung inflammation during LPS‐induced acute lung injury during obesity. This adds NOD1 as an innate immune receptor that exerts beneficial and compartmentalized effects on pulmonary inflammation during acute lung injury (E‐Lacerda et al., 2020; Rodrigues E‐Lacerda et al., 2024). Importantly, most of the inflammatory phenotypes associated with NOD1 deficiency, including elevated cytokine production (BAL and lung), impaired neutrophil clearance, and altered macrophage and inflammatory markers, were observed only in obese mice. In animals under a control diet, NOD1 deficiency did not significantly alter BAL cell counts, lung cytokines, gene expression profiles, or senescence markers. This strongly suggests that the inflammatory consequences of NOD1 deletion are context‐dependent, becoming apparent only under metabolic stress induced by a high‐fat diet. These findings support the notion that NOD1 acts as a protective regulator of pulmonary inflammation specifically during obesity‐associated immune dysregulation.

NOD1 deletion resulted in reduced neutrophil migration into the BAL, while maintaining elevated levels of different type 1 cytokines. These findings suggest that NOD1 appears to be involved not only in neutrophil recruitment but also in the retention and clearance of these cells from the lung parenchyma into the airspaces. Neutrophil clearance from the lungs is a tightly regulated process that involves mechanisms such as apoptosis and efferocytosis (Greenlee‐Wacker, 2016). An efficient resolution of pulmonary inflammation depends not only on the control of leukocyte recruitment but also on the removal of inflammatory cells from the parenchyma to prevent excessive lung tissue damage (El Kebir et al., 2012).

This observation is further supported when analyzed alongside the data from lung parenchyma inflammation, where NOD1 deletion in obese mice led to increased neutrophil recruitment and elevated type 1 cytokine production. The reduced number of cells in the BAL of obese NOD1‐deficient mice should not be initially interpreted as indicative of a pro‐inflammatory role for NOD1 signaling. In fact, when considering the increased leukocyte accumulation in the lung parenchyma of these mice, it becomes evident that NOD1 acts in facilitating the clearance of these inflammatory cells from the tissue to the alveolar space. The apparent dissociation between reduced neutrophil numbers in the BAL and elevated type 1 cytokine levels in obese Nod1^−/−^ mice further supports this interpretation. Rather than reflecting a lower inflammatory state, the decreased cellularity in the alveolar space likely results from impaired leukocyte clearance from the parenchyma. In the absence of NOD1, infiltrated cells may persist within lung tissue, sustaining cytokine production and contributing to an unresolved inflammatory environment. These findings reinforce the notion that NOD1 plays an active role in coordinating both leukocyte dynamics and the resolution of inflammation during obesity‐associated lung injury. We also observed increased SA‐β‐galactosidase activity in the lungs of obese NOD1‐deficient mice, suggesting the presence of senescent cells, which contribute to a sustained inflammatory environment through the senescence‐associated secretory phenotype (SASP), a process characterized by the release of pro‐inflammatory cytokines, chemokines, and matrix remodeling enzymes that can amplify and prolong tissue inflammation (Aoshiba et al., 2013; Huidobro et al., 2021).

Additionally, NOD1 deficiency in obese mice was associated with a reduction in CD163^+^ cells and an inflammatory transcript profile collectively suggesting a shift toward a more pro‐inflammatory, M1‐like macrophage phenotype. The phenotypic profile of pulmonary macrophages may also contribute to this imbalance in inflammatory cell dynamics. Acute lung injury is characterized by the presence of neutrophils and classically activated (M1) macrophages, which produce high levels of pro‐inflammatory mediators such as TNF‐α, IL‐1β, IL‐6, and reactive oxygen species (ROS) (Johnson & Matthay, 2010; Man et al., 2021). Resolution of inflammation, however, requires a phenotypic switch from M1 to alternatively activated (M2) macrophages, which play a key role in tissue repair. M2 macrophages help restore immune balance through the secretion of anti‐inflammatory cytokines such as IL‐10 and TGF‐β, actively participate in the efferocytosis of apoptotic neutrophils, and promote tissue remodeling through the controlled deposition of extracellular matrix components (Allard et al., 2018; Melo et al., 2021; Shen et al., 2019).

The concepts discussed above are further supported by the altered mRNA expression of key inflammatory genes in the lungs. Under obese conditions, NOD1 deletion led to increased expression of the chemokines *Cxcl9*, *Cxcl10*, and *Mcp1*, establishing a chemotactic environment that favors the recruitment and retention of inflammatory cells (Kameda et al., 2020). Elevated expression of *Itgam* and *Itgax* likely contributes to sustained leukocyte adhesion and activation, promoting cellular accumulation in lung tissue and impairing clearance (Hu et al., 2023). In addition, increased expression of *Cd40* reinforces antigen‐presenting cell activation, thereby amplifying local inflammation and potentially inhibiting M2 macrophage polarization (Hashimoto et al., 2004). We also observed increased expression of *Nlrp3* and *Casp1* in obese NOD1‐deficient mice, which may reflect increased NLRP3 inflammasome priming rather than inflammasome activation. Direct evidence of NLRP3 inflammasome assembly, activation, or caspase‐1 cleavage was not assessed. The NLRP3 inflammasome is a key driver of lung inflammation and has been implicated in several pulmonary conditions that share pathophysiological features with acute lung injury, including pneumonia and acute respiratory distress syndrome (ARDS) (Gu et al., 2024). Pulmonary injury is associated with various inflammasome‐related pro‐inflammatory and regulatory components, such as DAMPs, NLRP3, ASC, caspase‐1, gasdermin D, and effector cytokines like IL‐1β (McVey et al., 2021). The upregulation of *Nlrp3* and *Casp1* in our model may reflect a primed inflammatory environment in the absence of NOD1, potentially favoring inflammasome activation and contributing to persistent inflammation in the obese lung.

In addition to these findings, we demonstrated that obese NOD1‐deficient mice exhibited increased phosphorylation of NF‐κB p65 and ERK1/2, along with decreased phosphorylation of p38 MAPK, highlighting the role of NOD1 signaling in the regulation of these major inflammatory pathways. It is well established that NOD1 activates NF‐κB and MAPK signaling upon recognition of bacterial peptidoglycans (Rodrigues E‐Lacerda et al., 2023). However, we hypothesize that obesity‐induced modifications, combined with the absence of NOD1 itself, may lead to compensatory activation of alternative PRRs, as suggested by the increased pulmonary expression of *Nod2* observed in NOD1‐deficient mice. While the heightened NF‐κB and ERK1/2 signaling aligns with the pro‐inflammatory phenotype described by us, the reduced p38 phosphorylation in obese NOD1‐deficient mice remains more difficult to interpret. Under specific conditions, p38 activation can be initiated by pro‐resolution molecules such as annexins, which contribute to the resolution of inflammation by promoting cellular differentiation, apoptosis, and extracellular matrix regulation. In particular, annexin A1, through engagement of its receptor FPR2, can drive the production of IL‐10 and polarization of macrophages toward an anti‐inflammatory M2 phenotype (Filep, 2013; Yan et al., 2022). Although this pathway would be consistent with the M2 macrophage profile observed in our study, the pulmonary gene expression of *Anxa1*, *Fpr2*, and *Casp3* in NOD1‐deficient mice was not significant or showed changes that were not aligned with this anti‐inflammatory signature. Thus, while alterations in p38 signaling may contribute to the inflammatory imbalance observed, the exact mechanisms underlying its regulation in the context of NOD1 deficiency and obesity‐associated lung injury warrant further investigation.

A limitation of this study is the use of non‐littermate control mice. Although the Nod1^−/−^ mice were backcrossed for over 12 generations into the C57Bl/6N background and age‐matched controls were obtained from the same source, we recognize that littermate controls would provide a more genetically rigorous comparison. Nevertheless, the observed phenotypes were consistent, biologically plausible, and aligned with known roles of NOD1 in immune‐metabolic regulation (Correa et al., 2012; Schertzer et al., 2011). Future studies using conditional and littermate‐controlled models will be valuable to extend these findings.

## 5 CONCLUSION

Together, these findings demonstrate that NOD1 plays a critical role in modulating LPS‐induced acute lung injury during obesity. NOD1 deficiency altered neutrophil dynamics, sustained type 1 cytokine production, disrupted macrophage polarization, and led to dysregulation of NF‐κB, ERK, and p38 MAPK signaling pathways. These findings provide novel insight into the immune‐metabolic regulation of lung injury and highlight NOD1 as a potential target for modulating inflammation in obesity‐related respiratory diseases.

## AUTHOR CONTRIBUTIONS

Conceived and designed research by G.F.A., J.D.S., and R.R.L.; performed experiments by R.R.L., N.G.B., and H.F.; analyzed data and interpreted results of experiments by R.R.L. and J.D.S.; prepared figures, drafted manuscript, edited and revised manuscript, and approved final version of manuscript by R.R.L., N.G.B., H.F., G.F.A., and J.D.S.

## FUNDING INFORMATION

The Sao Paulo Research Foundation (FAPESP, Brazil), grants 2019/03751‐3 and 2021/09365‐8. Natural Sciences and Engineering Research Council of Canada, grant RGPIN‐2020‐05707.

## CONFLICT OF INTEREST STATEMENT

The authors declare no competing interests.

## ETHICS STATEMENT

All procedures were approved by the McMaster University Animal Research Ethics Board (AREB) and conducted in accordance with the Canadian Council on Animal Care (CCAC) guidelines.

## Supporting information


Figure S1.

## Data Availability

https://data.mendeley.com/preview/tkwnhzxfh6?a=ce82fd71‐1e09‐409f‐bfca‐e37c30ac2c70.
