# Supplementary material for: NOD1 promotes leukocyte clearance and limits inflammation in female mice during obesity‐associated acute lung injury
Source: Physiol Rep. 2025 Jul 4;13(13):e70446. doi: 10.14814/phy2.70446 (PMC12227658; doi:10.14814/phy2.70446)
Supplement: Supplementary file 1 — Figure S1. [file PHY2-13-e70446-s001.docx]

**SUPPLEMENTAL FIGURE 1**


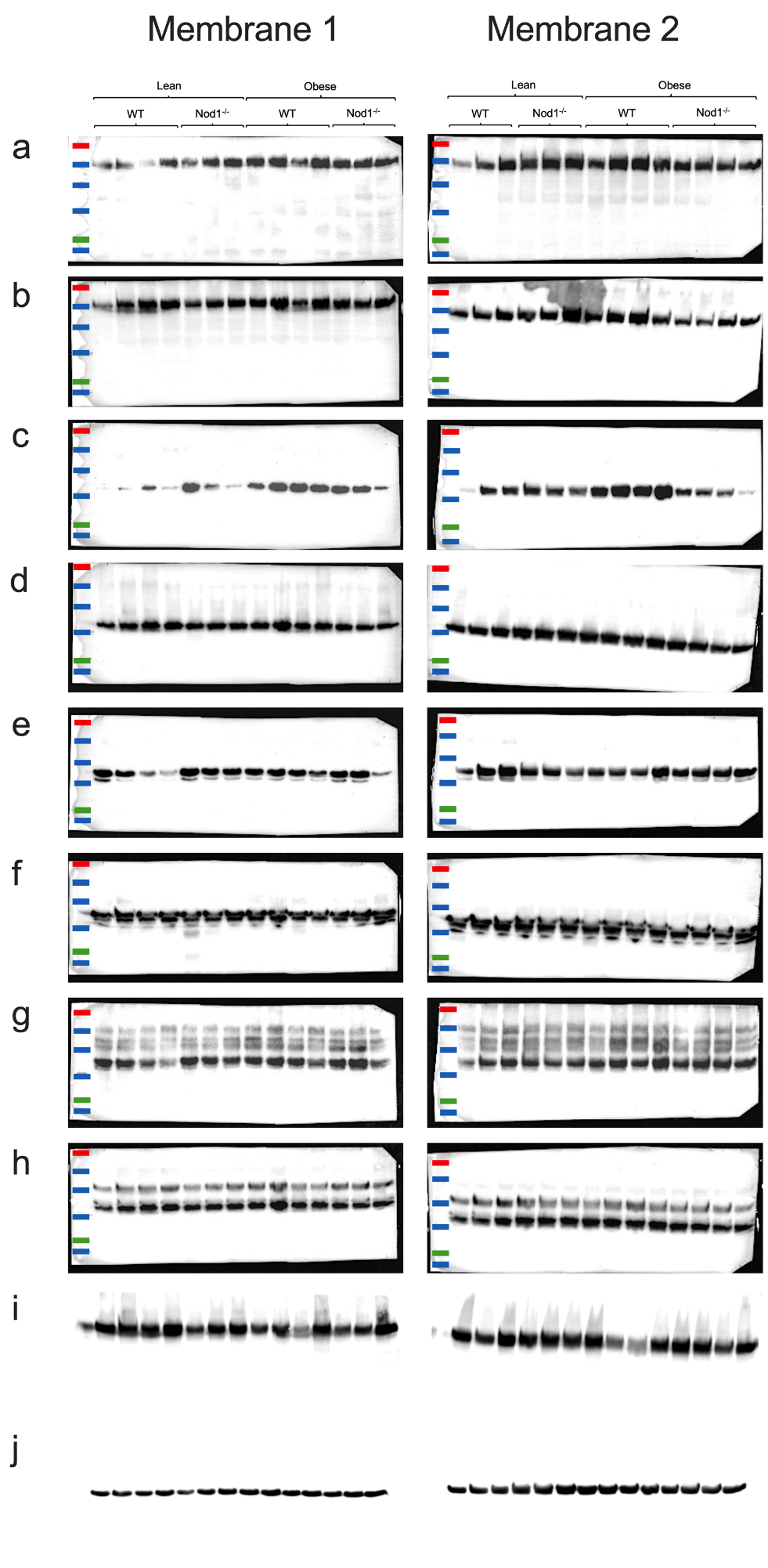


**Supplemental Figure** **1. Uncropped Western blot membranes for all analyzed proteins.** (a) NF-κB p65. (b) phospho-NF-κB p65. (c) p38 MAPK. (d) phospho-p38 MAPK. (e) ERK 1/2. (f) phospho-ERK 1/2. (g) JNK. (h) phospho-JNK. (i) MPO. (j) β-Actin. Protein ladder from top to bottom (kDa): 75 (red), 63 (blue), 48 (blue), 35 (blue), 25 (green) and 20 (blue). [https://doi.org/10.6084/m9.figshare.29047529.v1](http://dx.doi.org/10.6084/m9.figshare.29047529.v1)
